# SEM Analysis of Glued Joints of Thermally Modified Wood Bonded with PUR and PVAc Glues

**DOI:** 10.3390/ma15186440

**Published:** 2022-09-16

**Authors:** Miroslava Mamoňová, Dávid Ciglian, Ladislav Reinprecht

**Affiliations:** 1 Department of Wood Science, Faculty of Wood Sciences and Technology, Technical University in Zvolen, T. G. Masaryka 24, 960 01 Zvolen, Slovakia; 2 Department of Wood Technology, Faculty of Wood Sciences and Technology, Technical University in Zvolen, T. G. Masaryka 24, 960 01 Zvolen, Slovakia

**Keywords:** Norway spruce wood, thermal modification, polyurethane glue, polyvinyl acetate glue, scanning electron microscopy, glued joint, glue line, penetration depth

## Abstract

This study deals with the scanning electron microscopy (SEM) analyses of the phase interfaces in the glued joints between Norway spruce wood elements thermally modified at 160, 180, 200, and 220 °C/4 h and polyurethane (PUR) and polyvinyl acetate (PVAc) glues with the aim of evaluating various anatomical influences of wood on the quality of joints. Due to cracked regions created in the surface of spruce wood at severe thermal modifications, the penetration depth of glues doubled from 140 to 241 μm for PUR glue, and from 100 to 200 μm for PVAc glue. The thickness of glue lines in joints was apparently higher for PVAc glue, mainly in earlywood regions; however, in joints from thermally modified (TM) woods it increased more apparently for PUR glue from 16.6 to 44.4 μm, probably in connection with formation of micro-bubbles in its structure. The SEM analyses corresponded well with the previous knowledge about lower shear strength of glued joints formed from the more intensively TM spruce elements and mentioned types of glues.

## 1. Introduction

Currently, thermally modified (TM) wood is widespread in various products used in the interior, such as bathrooms, saunas, and kitchens, as well as in the exterior, especially for structures without ground contact, such as windows, front doors, facades, gazebos, pergolas, garden furniture, or sometimes in the form of terrace boards. According to the standard EN 335 [1], the products from TM wood belong to the 2nd up to the 4th lumber grade with dependence on the wood species and technological conditions of wood modification—temperature, time, pressure, and heating medium. TM wood is more resistant to biological agents compared to solid wood [2,3,4], and it has better dimensional stability and lower hydrophilicity [5,6]. Due to these beneficial properties, TM wood is preferred for use in environments with higher humidity to effectively replace rare tropical tree species [7,8]. However, besides its advantages, such as enhanced durability, increased dimensional stability, and higher hydrophobicity, its disadvantages include some of its mechanical properties, mainly impact bending strength and tension, depending on the technological parameters and tree species [5,9,10,11,12]. Even more disadvantageous, in some cases, are its gluing properties, needed for example, in the manufacture of glued laminated timber (glulam) beams. Therefore, TM wood is not usually recommended for construction elements, e.g., trusses, rafters, and other load-bearing items [13].

The structural arrangement of TM wood occurs firstly in its molecular chemical level, usually at temperatures ranging between 160–260 °C, especially connected with partial degradation of hemicelluloses, cleavage of hydroxyl groups from hemicelluloses, and lignin, and 3-D spatial crosslinking of hemicelluloses with thermally highly stable lignin [14,15,16]. Generally, hemicelluloses in Norway spruce wood, but also in other tree species, show lower thermal stability than cellulose due to lack of a crystallinity portion and lower polymerization degrees [17,18]. In a study by Vidholdová et al. [4] TM spruce wood lost individual hemicelluloses in this order: arabinose > mannose~glucose  >  xylose~galactose due to their depolymerisation, creation of internal ethers, and other rearrangement products, and finally, decomposition into volatile compounds [18,19,20]. Changes in the molecular level of TM wood have also been observed through infrared spectroscopy [21,22], and indirectly through colour changes [23,24], or creation of splits and cracks [25,26]. Details of the brittle behaviour of TM wood were revealed by means of images of fracture surfaces at the ultrastructural level. The changes in the molecular structure of TM wood are reflected by more or less important changes in its anatomical structure and mechanical properties [27]. Brittle cell walls of xylem can be more easily broken down due to mechanical loading, whereby the fracture area is smoother and less fibrous. The highest intensity of changes can be found in surface areas of wood exposed to a radiant or contact heat source [8].

All mentioned structural changes in TM wood can result in an increase of its surface hydrophobicity and roughness [28,29,30], and subsequently, in changes in gluing quality. Widmann et al. [31,32] dealt with the quality assessment of glulam beams and finger-jointed boards made out of TM wood, at which the lowest strength of glued joints between individual lamellas was manifested in the greatest weakness of glued trusses prepared from thermally modified lamellas. In a study by Dilik and Hiziroglu [33], the adhesion strength of glued joints formed from juniper wood thermally modified at temperatures of 120, 160, and 190 °C for 6 h and glued with polyvinyl acetate (PVAc) glue was decreased as a result of more intensive thermal modification processes. An evident decrease in the strength of glued joints of 23.6% occurred when the wood was modified at a relatively low temperature of 120 °C, while the most significant decrease of 64.1% occurred in the case of wood modified at a maximum temperature of 190 °C, due to the most apparent changes in its structural levels. The experiments carried out by Vidholdová et al. [34] also showed a significant decrease in the shear strength of glued joints between lamellas of spruce wood thermally modified at temperatures of 160 to 220 °C for 4 h, and bonded with polyurethane (PUR) and PVAc glues. When wood was modified at a temperature of 220 °C, there was a 56.1% decrease in the strength of glued joints in the case of PUR glue application and a 42.3% decrease in joint strength when PVAc glue was applied. Kol et al. [35] found a lower adhesion strength of TM spruce wood prepared at a temperature of 212 °C for 2 h and bonded with various types of glues suitable for glulam and other wood products, in comparison to similarly bonded unmodified spruce wood. However, the effect of the specific type of glue on a decrease in adhesion strength was not found. The authors of other studies draw the same conclusions that the strength of glued joints from TM wood is lower, whereby the level of a decrease in the strength does not depend predominantly on the type of glue used, but largely depends on the tree species used and also the technological parameters of thermal modification, such as temperature, pressure, and time [36,37,38]. Changes in the pH value of the TM wood surface might retard or accelerate the curing of glues, depending on their type [39]. Findings of the previously mentioned authors were confirmed also by the study of Boruszewski et al. [40], which investigated the effect of thermal modification of beech and birch wood at the temperatures of 160 and 190 °C on the strength of glued joints formed by means of melamine-urea-formaldehyde (MUF) resin, melamine-formaldehyde (MF) resin, and PUR glue. Results showed that TM wood can be effectively bonded using commercially produced PUR glue, as well as water solutions of MUF and MF resins, whereby the strength of glued joints is limited only by the strength and surface characteristics of the TM wood.

Scanning electron microscopy (SEM) studies on changes in the anatomical structure of the TM wood heated at 170 and 200 °C, showed that the greatest damage happened in the thinner areas of cell walls in the case of coniferous tree species, especially in pit membranes in the cross-field pits connecting longitudinal tracheids with radial ray parenchyma cells [41]. This damage is believed to play an important role in explaining the differences in water absorption between pine and spruce woods since the piceoid cross-field pits in spruce wood seemed to be unaffected by heat treatment [41]. The same conclusions were confirmed in the SEM study carried out by Awoyemi et al. [42]. In terms of changes in Norway spruce wood at the cellular level, drying in a reduced oxygen atmosphere at temperatures up to 220 °C has been found to cause deformation in the earlywood and tangential intracellular cracks in the latewood [43]. In the case of thermal modification of western red cedar (*Thuja plicata*) wood, destruction of tracheid cell walls, ray tissues, and pit deaspiration occurred at a temperature of 220 °C. It is evident that the brittleness of cell walls of xylem is a limiting factor for the preparation of cuts from the thermally modified coniferous as well as broad-leaved tree species, especially in transversal cuts, and such were prepared also for SEM observations in this work. Better findings related to wood structure can be obtained when the structure of TM wood is analyzed in situ using an environmental scanning electron microscope (ESEM) equipped with a hot stage [44,45,46,47,48].

Other studies also revealed that thermal modification of woods causes changes in their anatomical structure, which are strongly associated with the wood species and the characteristics of the modification process [49]. For example, interesting conclusions are mentioned for Douglas fir by Boonstra et al. [50] when two-stage heat treatment under 200 °C did not cause damage to the ray parenchyma, as well as in pit membranes, bordered pits, and large window pit membranes, at which the margo fibrils also appeared without damage, such that, compared to the other coniferous species tested, it had the most stable structure at heat treatment.

The aim of this study was to microscopically analyze the phase interfaces of Norway spruce wood modified at various temperatures and glued with PUR or PVAc glue, which are important for the final quality of glued joints. It follows the experiments of Vidholdová et al. [34], which showed that the shear strength of glued joints of TM spruce woods decreased with rise in modification temperatures. The results of this study could further advance the understanding of the principles of gluing TM wood, and creation of the phase interface of thermally modified wood glue.

## 2. Materials and Methods

### 2.1. Wood and Thermal Modification of Wood

Five defect-free boards of Norway spruce (*Picea abies* Karst. L.) wood with a length of 40 cm and a width of 25 cm were machine-milled to a thickness of 1 cm, and then were used in the experiment. Initially, the boards were dried at 100 °C ± 3 °C to the oven-dry state, and subsequently, four of them were thermally modified at 160 °C, 180 °C, 200 °C, or 220 °C for 4 h at atmospheric pressure in a drying oven (Memmert UFE 500, Schwabach, Germany).

From the reference and four TM boards specimens were prepared with the dimensions of 80 mm × 20 mm × 5 mm (longitudinal × radial × tangential). No knots, splits, checks, or other non-homogeneity occurred in the specimens. Deflection between the growth rings and the cross-sectional areas of the specimens ranged from 30° to 90°. Finally, the areas determined for gluing were sanded using 120-grit sandpaper.

All wood specimens were conditioned at 20 °C ± 2 °C and 65% RH for 21 days to achieve equilibrium moisture content before their gluing, which ranged from 6.7 to 11.8%, depending on the modification temperature, i.e., 11.8% for reference unmodified wood and 6.7% for wood thermally modified at 220 °C. Density of the sound spruce wood was 0.459 ± 0.018 g/cm^3^ and reduced to 0.426 ± 0.015 g/cm^3^ for wood TM at 220 °C.

### 2.2. Glues

Two types of glues were used: (a) one-component PUR glue Kestopur 1030 (Kiilto Oy, Tampere, Finland), and (b) one-component PVAc glue Rakoll^®^ 4330 (H.B. Fuller Europe, Zürich, Switzerland). The parameters of glues used are mentioned in Table 1.

### 2.3. Preparation of Glued Joints

The selected glue types were applied on the smoothed surfaces (80 mm × 20 mm) of the spruce wood specimens at a recommended rate of 180 g/m^2^. Bonding of specimens was performed in steel bolts at a pressure of 1.2 MPa, a temperature of 20 °C ± 2 °C, and a relative humidity of 65% ± 5%, for 2 h. The following testing sets were formed from a non-modified (100 °C) and the thermally modified specimens (160 °C, 180 °C, 200 °C, or 220 °C), i.e., these combinations were formed: (I) 100 °C/100 °C ⇒ one reference testing set, (II) 160 °C/160 °C, (III) 180 °C/180 °C, (IV) 200 °C/200 °C, (V) 220 °C/220 °C ⇒ four thermally modified testing sets, (VI) 100 °C/220 °C ⇒ one combined testing set. These testing sets were prepared by using PUR and PVAc glue.

### 2.4. Microscopic Analysis of Glued Joints

**Figure 1:**
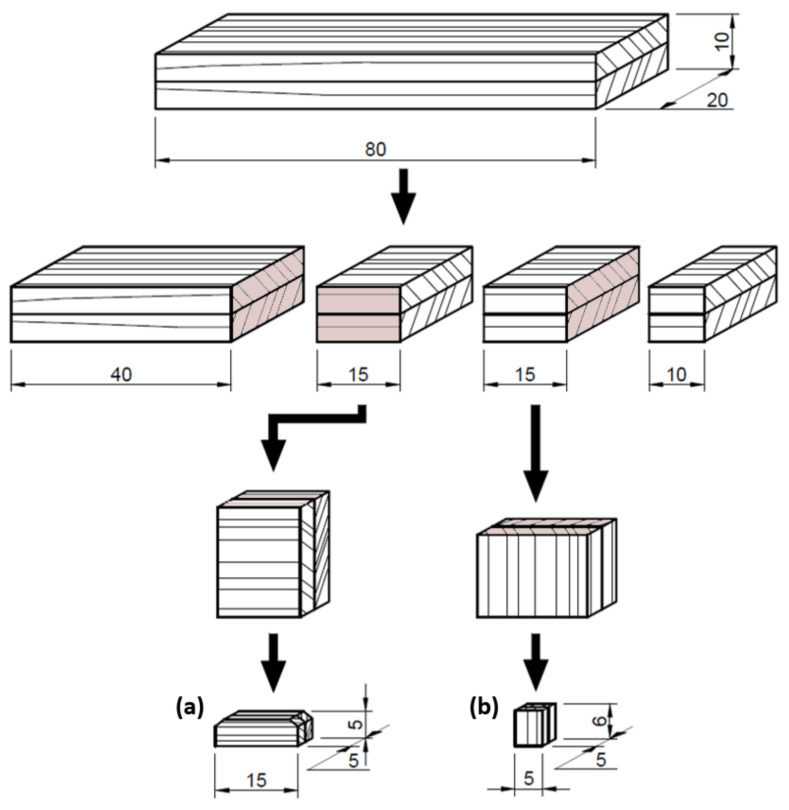
Preparation of the mounting specimens with longitudinal (**a**) and cross-sectional (**b**) cuts of glued joints for SEM analysis.

The glue lines were analyzed both along and across the grain direction. The mounting specimens were selected after cutting from the given testing specimen sets. The longitudinal surface of the mounting specimens with the dimensions of (a) 15 mm × 5 mm × 5 mm (longitudinal × radial × tangential) and their cross-sectional surface with dimension of (b) 6 mm × 5 mm × 5 mm (longitudinal × radial × tangential) were subjected to SEM analysis (Figure 1).

The cutting of specimens for SEM analysis was conducted in a fixing bio-holder to decrease the fragility of the surface layer of the TM wood and to achieve an ideal cut [51,52].

**Figure 2:**
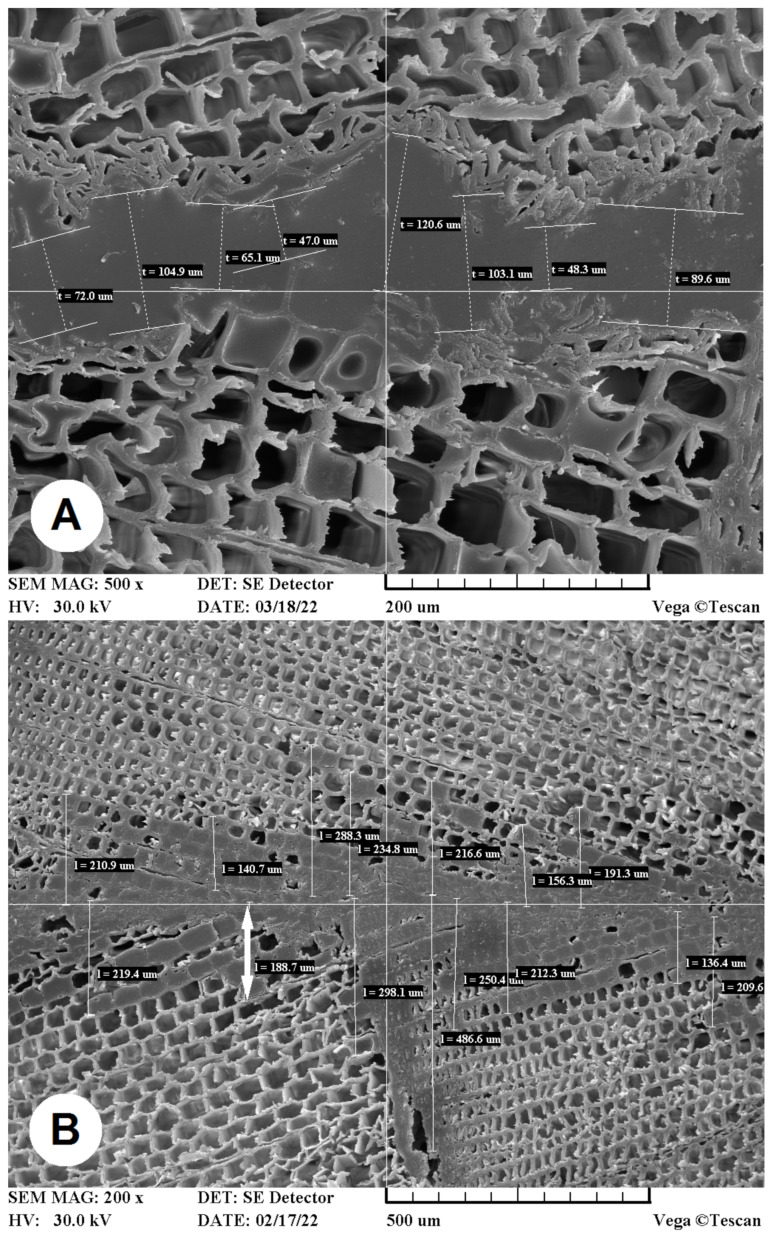
The methodology of recording (**A**) the thickness of the glue line of PVAc glue for testing set IV = 200 °C/200 °C, and (**B**) the penetration depth of PUR glue into the structure of TM spruce wood (highlighted with white arrow) for testing set V = 220 °C/220 °C.

The mounting specimens with glued joints of TM wood were attached to the aluminum stubs using conductive carbon adhesive tabs (20 mm) and the circumference was covered with a conductive colloidal silver paint, sputter-coated with gold in device K650X (Quorum Technologies Ltd., Laughton, UK), and examined with high-vacuum scanning electron microscopy using a Tescan Vega SEM (Tescan, Brno, Czech Republic) operating at 30.0 kV. The electron source was a tungsten filament. The operating conditions, including magnification and scale bar, were recorded on the data bar at the foot of the SEM image, as seen in Figure 2.

Upon determination of the depth of glue penetration into the structure of reference and TM spruce wood specimens, as well as the thickness of glue line, the measurement procedures for analysing the recorded images of cross-section cuts of mounting specimens were observed and evaluated (Figure 2).

The glue line thickness determinations were recorded as the shortest distance between the contours of two uneven surfaces formed by two cut tracheids. These measurements were recorded with an enlargement of 500× (Figure 2A).

The methodology for measuring the penetration depth of PVAc or PUR glue into the structure of TM spruce wood specimens is shown in Figure 2B. The centre of the glue line was positioned in the centre of the recorder image. Thus, the farthest lumen of the tracheid filled with PVAc or PUR glue was recorded and the shortest, perpendicular distance in μm was determined. These measurements were recorded with an enlargement of 200×.

## 3. Results and Discussion

The SEM analysis proved that the glued joints created from spruce wood modified at different temperatures and PUR or PVAc glue had characteristic anatomical and morphological manifestations of the glued lines. Effective interlocking of wood tissues by a glue, involving its penetration into all accessible micro- and nano-pores of wood along the glue line, is even more crucial for optimal adhesive performance where mechanically weakened wood surfaces, such as those of TM wood, have to be stabilized and strengthened.

**Figure 3:**
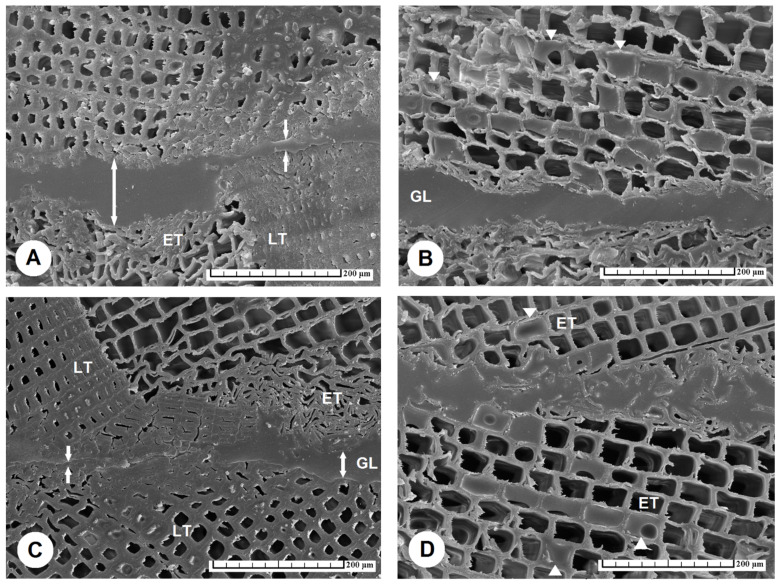
A glued joint of spruce TM wood with PVAc glue has a glue line (GL) with a large and uneven thickness, showing: (**A**) a significant difference in the thickness of GL for earlywood tracheids (ET) and latewood tracheids (LT) recorded for TM wood prepared at 220 °C, (**B**) the penetration depth of PVAc glue into the lumens of ET of TM wood prepared at 220 °C, with arrows showing the farthest-filled lumens of tracheids, (**C**) the difference in the thickness of GL observed also in the case of TM wood prepared at 180 °C, whereby significant effects occurred in the area of ET, and (**D**) TM wood prepared at 200 °C, in which the penetration depth of PVAc glue into ET is demonstrated by white arrows pointing to the farthest-filled lumens of tracheids. Scale bar = 200 μm. Abbreviations: ET—earlywood tracheids, LT—latewood tracheids, GL—glue line.

**Figure 4:**
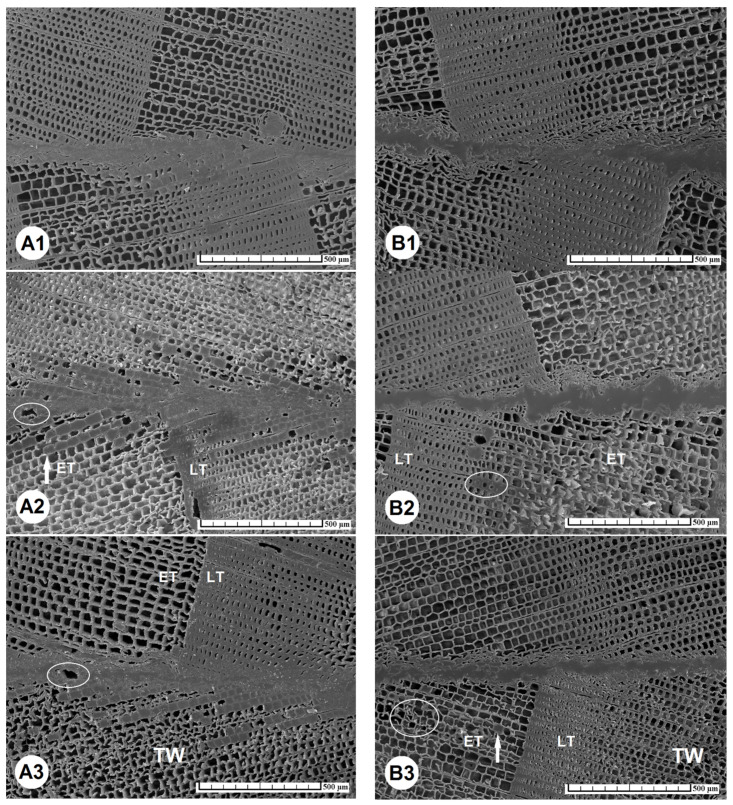
The effect of modification temperature on the quality of glued joints using PUR (**A1**–**A3**) and PVAc (**B1**–**B3**) glues was analyzed on the cross-section cuts. (**A1**,**B1**) show the thermally unmodified specimens—the reference testing set; (**A2**,**B2**) the thermally modified testing set (V) 220 °C/220 °C; and (**A3**,**B3**) the combined testing set (VI) 100 °C/220 °C from the reference specimen in the upper part of the image and the ThermoWood specimen in the lower part of the image. Scale bar = 500 μm. Abbreviations: ET—earlywood tracheids, LT—latewood tracheids, TW—thermally modified wood.

The surface of TM wood is characterized by open lumens of longitudinally sectioned tracheids, the cell walls of which are brittle and subject to plastic deformation up to collapse, observable on cross-sections as a cracked region (Figure 3A–D). Our observations are consistent with the conclusions of Singh et al. [45], who found that the glue penetrated a few cells deep into tracheids, but more deeply into cracked regions (Figure 3 and Figure 4).

In addition to the chemical molecular adhesive bonds that are a precursor to forming a strong bond in one-component polyurethane (PUR) or polyvinyl acetate (PVAc) glues, the fragility of the surface layers of the TM wood plays an essential role in the formation of the adhesive bond, thus allowing penetration of the glue into the deeper layers of the wood.

The course of the glue line of the reference specimens showed that it was narrow and continuous in the case of the PUR glue (Figure 4A1) (average value 16.6 μm, Table 2), compared to the PVAc glue (Figure 4B1), where a meandering course occurred, with the greatest thickness of the glue line observed in the region of the earlywood tracheids, up to 76 μm (average value 49.3 μm, Table 2). Generally, the thickness of the glue line increased with the temperature of thermal modification of the spruce wood (Table 2).

A significant difference in the thickness of the glue line was recorded in the case of TM spruce wood prepared at 220 °C in the area of earlywood tracheids, when in some cases the thickness of the glue line was >100 μm (Table 2), whereby, in the case of latewood tracheids the thickness of the glue line was only ~20 μm (Figure 3A).

The penetration depth of the glue into the structure of TM spruce wood increased with increase in temperature. A significant difference was observed for the reference specimens, where the PUR glue penetrated to a depth of 139.8 μm and the PVAc glue to 99.6 μm. In the case of TM wood prepared at 220 °C, the penetration depth was the highest; for the PUR glue, penetration reached a maximum depth of 514 μm with average penetration value of 240.9 μm, and for the PVAc glue maximum penetration of 262 μm was reached, with average penetration value of 200.3 μm (Table 2). Such high penetration depths into the TM spruce wood are due to the creation of tangential micro-cracks in the area of the latewood tracheids, where some lumens were filled with PUR glue (Figure 4A2).

**Figure 5:**
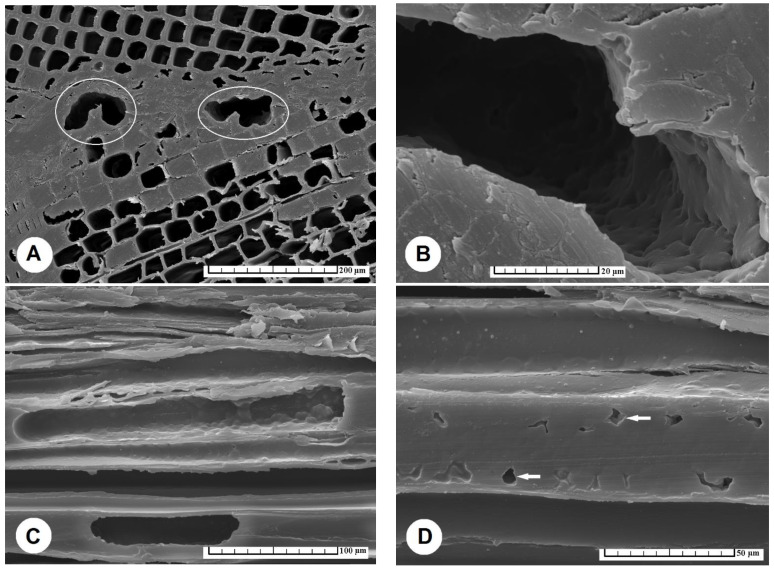
In the joints of TM spruce wood with PUR glue are seen typical micro-bubbles, including (**A**) irregular bubbles (100–150 μm, white ellipses) formed in the glued joint of the combined testing set VI 100 °C/220 °C, (**B**) a detail of the structure of the inner surface of bubbles in the cross-section cut of the glued joint, (**C**) a detail of the longitudinal shape of bubbles (100–200 μm) in the lumens of ET of TM wood (220 °C) on the longitudinal cut of the glued joint, (**D**) a detail of forming small micro-ruptures with the dimension of ~5 μm (white arrows) in the PUR glue coat on the S3 layer of cell wall. Scale bars = 20 to 200 μm.

The joints of TM spruce wood with PUR glue were characterized by the formation of micro-bubbles (Figure 4A2,A3 and Figure 5A; bubbles are highlighted by ellipses), as an indispensable consequence of a chemical reaction of one-component PUR glue with bound water in cell walls of tracheids.

The occurrence of micro-bubbles (Figure 5B,C) and small circumscribed micro-cracks in the PUR glue on the S3 layer of the earlywood tracheid (Figure 5D) may be the cause of a lower mechanical strength of this glue type, because the created micro-bubbles act as a pathway of weakness within the cured glue, as shown by the results of previous experiments [34,53,54,55].

The PUR glue penetrated the radial rows of the earlywood tracheids, thus forming the characteristic tree-like pattern (Figure 4A2). Because there were also rows of tracheids with lumens without glue among the patterns, we assume that glue penetration was mainly by capillary effect. In addition to the capillary effect, a deep penetration of PUR glue into the lumens of latewood tracheids of TM spruce wood consists in possible pathways through non-aspired pits on radial walls (Figure 4A2), while the permeability of the more numerous bordered pits of earlywood tracheids can be significantly limited due to thermal modification when the bordered pit apertures are totally closed [52,56,57,58].

For the combined testing set VI (100 °C/220 °C), there occurred the brittle structures and higher asymmetric penetration of the PUR glue into the TM wood as into the reference unmodified wood (Figure 4A3 and Figure 5A). Penetration of the PVAc glue into the deeper structure of the TM wood was observed only in the lumens of the earlywood tracheids (Figure 4B3).

**Figure 6:**
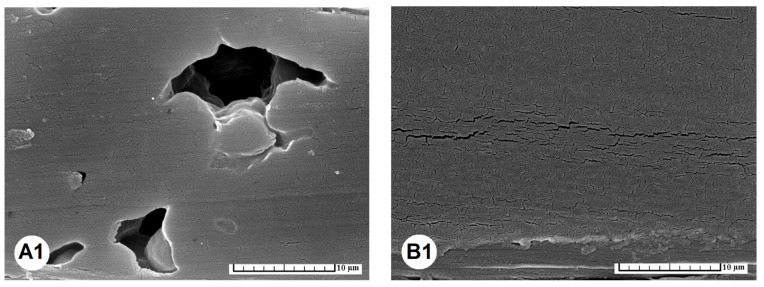

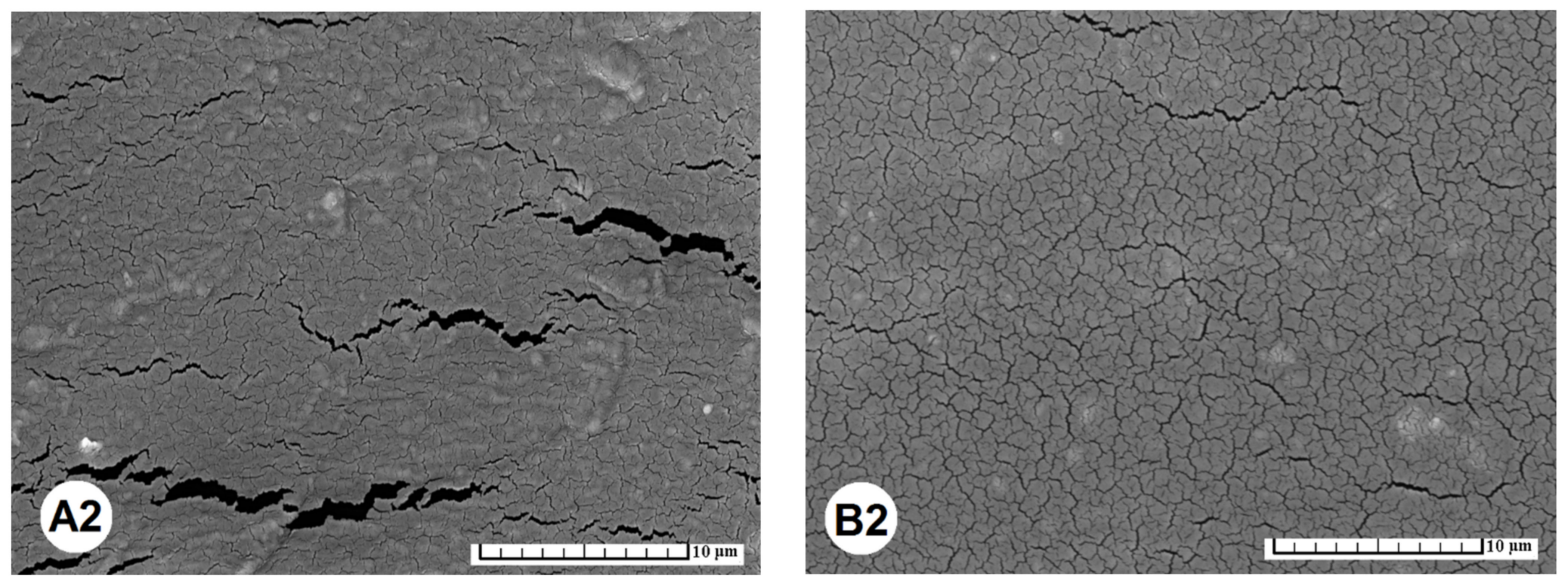
Morphology of the structure of glued joints using PUR (**A1**,**A2**) and PVAc (**B1**,**B2**) glues for bonding the TM spruce wood prepared at 220 °C. In the PUR glue typical micro-bubbles were formed and seen in the longitudinal cut (**A1**), while in the cross-section cut exceeding fragility of the glue layer occurred (**A2**). A seemingly smooth line of a joint created from PVAc glue shows fragility when enlarging by 8000× in the case of longitudinal cut (**B1**) as well as cross-section cut (**B2**), and it is finer in comparison to PUR glue. Scale bar = 10 μm.

SEM structural analysis of glued joints of the TM spruce wood prepared at 220 °C (Figure 6) showed that the seemingly smooth joint line with PVAc glue exhibited fragility when enlarged by 8000×. It is finer in comparison to the PUR glue line in the case of longitudinal (Figure 6B1) as well as cross-section cut (Figure 6B2). Formation of uneven micro-bubbles was typical for the PUR glue, well-shown in the longitudinal cut (Figure 6A1). In the cross-section cut (Figure 6A2) exceeding fragility occurred.

Previous research by Vidholdová et al. [34] has demonstrated a decrease in the shear strength of glulam elements when spruce boards were prepared from TM timber at increased temperatures from 160 to 220 °C. A higher reduction in the shear strength was reported with the application of the PUR glue (maximally about 56.1%) than with the PVAc glue (maximally about 42.3%). This result can be explained by these additional factors: (1) changes in the thickness of the glue line; (2) changes in the depth of glue penetration in connection with creation of cracks in TM wood surfaces; (3) increased hydrophobicity and worsened wettability of TM wood surfaces; (4) creation of micro-bubbles in the layer of PUR glue contacting more cracked, porous, and hydrophobic surfaces of the thermally modified spruce wood. Factor (4) is the most acceptable hypothesis and it is also in accordance with presented results of the SEM studies performed in this work on similarly prepared glued joints.

## 4. Conclusions

(1) For better understanding of the effectiveness of a glued joint in glued beams, it is very important to be familiar with the interaction between the adherent and the glue/adhesive. The scanning electron microscope is a tool that can help us to understand why the mechanical interlocking of glue into wood tissues is considered important in adhesive performance and strength of glued joints.
(2) The microscopic analysis showed that the many benefits of thermally modified Norway spruce wood can be eliminated by the detriments related to well-known changes in the molecular structure, but also to following changes in the anatomical and morphological structure, especially reflected in the fragility of the cell walls and the formation of cracked regions.
(3) The increase in brittleness and creation of micro-cracks in spruce wood modified at higher temperatures implies the possibility of glue penetration into the deeper layers of this wood species.
(4) Increased penetration depth into the cell lumens of the thermally modified spruce wood tracheids was already marked at lower modification temperature of 180 °C for polyvinyl acetate (PVAc) glue; however, for one-component polyurethane (PUR) glue only at the highest temperature of 220 °C.
(5) The thickness of the glue lines increased for testing sets consisted of specimens thermally modified at higher temperatures from 160 to 220 °C.
(6) Micro-bubbles created in the glue lines of PUR glue, more evidently in contact with TM wood surfaces, could be a potential for weakness of glue joints in glulam beams or other glued products prepared from thermally modified woods.

## Figures and Tables

**Table 1:** Physical and chemical characteristics of glues used.

Glue	Kestopur 1030	Rakoll^®^ 4330
Type	Polyurethane (PUR)	Polyvinyl acetate (PVAc)
Viscosity at 20 °C [mPa·s]	7000	13,000
Density [kg/m^3^]	1200	1100
Color	Transparent, light after drying	White, transparent after drying
pH value	–	3
Recommended spread rate [g/m^2^]	160–200	160–180
Open time [min]	30	8–12
Pressing time [min]	90–120	10–15

**Table 2:** The thickness of glue line and the penetration depth of PUR and PVAc glues into the structure of reference and thermally modified spruce wood specimens.

Type of Joint-Testing Set Specimen	Thickness of Glue Line (μm)			Penetration Depth of Glue into Wood Structure (μm)		
	Average (SD)	Max	Min	Average (SD)	Max	Min
**PUR glue**						
100 °C/100 °C (Reference)	16.6 (6.4)	33	8.5	139.8 (78.1)	378	54
160 °C/160 °C	26.1 (8.7)	43	13	157.0 (59.3)	260	85
180 °C/180 °C	31.7 (2.7)	35	29	170.8 (46.8)	295	76
200 °C/200 °C	32.1 (7.6)	53	21	180.0 (111.6)	460	97
220 °C/220 °C	44.4 (16.0)	77	24	240.9 (82.5)	514	136
**PVAc glue**						
100 °C/100 °C (Reference)	49.3 (17.3)	76	20	99.6 (56.3)	270	48
160 °C/160 °C	55.3 (26.2)	91	19	115.8 (41.5)	214	81
180 °C/180 °C	67.3 (18.3)	92	36	186.7 (67.1)	310	82
200 °C/200 °C	74.8 (31.7)	140	22	189.0 (86.5)	402	63
220 °C/220 °C	66.9 (25.2)	120	16	200.3 (42.0)	262	138

Note: Average—mean values of the penetration depth are related to the highest depth of glue penetration into the crack regions of the TM wood specimens; SD—standard deviation. The number of measured values of thickness of glue line for each joint-testing set (JTS), *n* = 25; the number of measured values of penetration depth of PUR glue, *n* = 30–40, and the number of measured values of penetration depth of PVAc glue, *n* = 20–40, for each (JTS).

## Data Availability

Not applicable.
